# A multidisciplinary group-based survivorship intervention for those living with multiple myeloma: a feasibility study

**DOI:** 10.1186/s40814-024-01524-1

**Published:** 2024-07-15

**Authors:** Nora Eilert, Norma-Jean Murphy, Hannah Cummins, Emma Houlihan, Janusz Krawczyk

**Affiliations:** 1 Cancer Care West, 72 Seamus Quirke Rd, Galway, Ireland; 2 Galway University Hospital, Newcastle Rd, Galway, Ireland; 3 University of Galway, University Rd, Galway, Ireland

**Keywords:** Multiple myeloma, Survivorship, Self-management, Exercise, Peer support

## Abstract

**Background:**

While multiple myeloma continues to be an incurable cancer, advances in its understanding and management have led to significantly improved survival rates. Survivorship interventions for those living with multiple myeloma remain scarce, despite mounting evidence for multiple unmet support needs among multiple myeloma survivors. The current study aimed to evaluate the feasibility and preliminary effectiveness of a novel multidisciplinary group-based multiple myeloma survivorship intervention.

**Methods:**

A mixed-method, repeated measures feasibility study was conducted within a routine cancer support service. Seven participants, aged over 18, who had a multiple myeloma diagnosis and were clinically assessed as suitable for the intervention by their haemato-oncologist, attended online for six weekly group sessions of physical exercise and self-management input, completing qualitative, physical and self-report measures at baseline, post-intervention and follow-up.

**Results:**

The intervention was deemed overall feasible, with relatively high uptake, participants describing it as largely acceptable and appropriate and providing recommendations for feasibility-enhancing intervention refinements. Findings regarding the preliminary effectiveness of the intervention were mixed. While qualitative analyses stressed the benefits of the intervention (e.g. peer support, connectedness, improved well-being) and large effect sizes were observed for most physical outcomes, no improvements in self-reported outcomes (i.e. quality of life, fatigue) were reported.

**Conclusions:**

This study represents the first investigation of a promising novel survivorship intervention for those living with multiple myeloma, highlighting the importance of peer support in particular, on which future clinical trials, aiming to establish the intervention’s effectiveness for routine care, will be able to build.

**Supplementary Information:**

The online version contains supplementary material available at 10.1186/s40814-024-01524-1.

## Key messages regarding feasibility

- What uncertainties existed regarding the feasibility?
  The ‘Living with Multiple Myeloma Group’ is a novel, group-based survivorship intervention for those living with multiple myeloma. This study represents the first implementation of the intervention and as such the feasibility of the referral pathway, of the intervention itself and of a definitive clinical trial to assess the effectiveness of the intervention were unclear prior to this study. Specifically, we did not know whether multiple myeloma survivors would sign up to take part in the intervention, would find its content, format and structure acceptable and appropriate or would perceive any positive effects from their participation in the intervention.

- What are the key feasibility findings?
  The intervention was found to be overall feasible. Intervention uptake was promising in respect of a larger trial and future routine referral pathways. Multiple myeloma survivors described the intervention as largely acceptable and appropriate and provided recommendations for feasibility-enhancing intervention refinements to inform future implementation of the intervention. The positive effects of the intervention reported qualitatively and via physical outcome measures were not sufficiently captured in self-report outcomes measures, raising questions regarding their appropriateness in being used in a larger trial.

- What are the implications of the feasibility findings for the design of the main study?
  This first implementation of the ‘Living with Multiple Myeloma Group’ confirmed the feasibility of the intervention on the level of the service user. The strengths of the study lie within the involvement of routine cancer care clinicians in the design of the research and intervention, promoting feasibility on the service provider and organisational levels also. The study’s findings meet the necessary requirements for the progression of the intervention into the next stage of intervention development, focused on effectiveness evaluations, e.g. via a pilot randomised controlled trial. Here, special consideration will need to be given to recommendations made by participants within this study and the careful selection of self-report outcome measures to ensure that the impacts of the intervention are appropriately captured.

## Background

Multiple myeloma (MM) is a haematological cancer affecting the plasma cells and thus often results in bone lesions, hypercalcemia, renal impairment, and anaemia. As such, common symptoms of MM include bone pain, fatigue, muscle weakness, breathing and gastrointestinal issues. The prevalence of MM has been rising steadily and has an age-standardised incidence rate of 2.1 per 100,000 persons worldwide now. While MM continues to be an incurable cancer, advances in the understanding and treatment of MM have led to better management of the illness (i.e. repeated periods of active treatment, chemotherapy, stem cell transplant and targeted treatments; interwoven with stretches of maintenance treatment), resulting in more patients achieving a minimal residual disease status and significantly improved survival rates [1–3] (e.g. survival has quadrupled across the UK in the last 40 years [4]).

Accordingly, those living with MM join the growing number of cancer survivors who do not get to ring the bell at the end of their treatment, declaring them cancer free and marking a return to normality, but rather have to live with ongoing symptoms and impairments associated with the cancer itself as well as the treatments they rely on to keep it at bay [3, 5, 6]. Research shows that about two-thirds of all cancer survivors have ongoing physical, psychological and supportive care needs after their primary treatment has ended and that these are often not adequately addressed by traditional care models [6–8]. Hence, new models of survivorship care have been advocated for, incorporating supported self-management and multidisciplinary rehabilitation interventions, aimed at optimising health, quality of life (QoL) and functioning and thereby addressing the needs of cancer survivors holistically and sustainably [8].

Research on self-management and rehabilitation interventions across oncology settings has found promising results; however, methodological shortfalls and heterogeneity of samples (i.e. tumour sites, cancer stage) limit generalisability to date [9–11]. In those living with MM specifically, the limited amount of research available has mostly focused on exercise interventions [9, 12–15]; despite strong evidence that MM survivors present with a multitude of unmet support needs, including physical (e.g. fatigue, pain) [16, 17], psychological (e.g. psychological distress, lack of peer support) [18–20] and educational needs (e.g. information regarding the disease and how to manage it) [21]. These unmet needs in turn have been shown to impact an individual’s QoL [3, 20, 22]. Early efforts in the development of multidisciplinary and self-management MM survivorship interventions have been scarce and found mixed results [14, 23, 24]. Furthermore, to our knowledge, no study has delivered a multidisciplinary intervention to MM survivors in a peer group format to date, despite peer support being desired by at least a subset of MM survivors, and potentially being particularly important in this cohort, given the impact MM may have upon personal relationships and social isolation [18, 20, 25].

Addressing this gap in the literature, the primary objective of the current study was to evaluate the feasibility (i.e., adoption, acceptability and appropriateness) of a newly developed multidisciplinary group-based survivorship intervention for those living with MM, the ‘Living with Multiple Myeloma Group’. The secondary objective of the study was to assess the preliminary effectiveness of the novel intervention via patient-centred outcomes. Implementing the intervention within a real-world setting and following guidelines for the development of maximally potent, implementable psychological and behavioural interventions [26, 27], the research further aimed to utilise feasibility, implementation and effectiveness data to generate recommendations for the adaptation and refinement of the intervention and inform upon future larger scale evaluations of the intervention.

## Method

### Design

A mixed-method, repeated measures feasibility study was conducted, using qualitative, physical and patient-reported outcome measures to evaluate primary feasibility and secondary preliminary effectiveness outcomes of the ‘Living with Multiple Myeloma Group’. Qualitative and quantitative findings were integrated at the interpretation stage for the purpose of triangulation. Feasibility evaluations were guided by Proctor and colleague’s implementation evaluation framework [28]. Reflecting the novelty and early-stage implementation of the intervention, feasibility was evaluated on the level of the individual MM survivor and for the purpose of this study conceptualised as adoption (i.e. uptake of the intervention by survivors), acceptability (i.e. satisfaction with the intervention) and appropriateness (i.e. perceived fit of the intervention with MM survivorship needs). Preliminary effectiveness was operationalised as physical improvements (aerobic capacity, hand grip strength, functionality of the lower body and fatigue) and psychosocial improvements (QoL, depression, anxiety and stress).

### Setting

The study was set in a not-for-profit, voluntary, community-based cancer support service, dedicated to providing professional, evidence-based, holistic support services (including psychological, exercise-based and nursing inputs) to cancer patients and their families in the West of Ireland. The study was conducted in conjunction with Galway University Hospital, one of nine designated cancer centres in Ireland, covering haemato-oncology services in West and North-West Ireland. Ethical approval for the study was obtained from the hospital’s clinical research ethics committee in June 2021 (reference: C.A.2646) and data collection took place between October 2021 and March 2022.

### Participants

Employing convenience sampling, Galway University Hospital patients were informed of the study and screened for eligibility by their consultant haematologist (5th author). Eligibility criteria were (1) being over 18 years old, (2) with a diagnosis of MM, and clinically assessed as (3) safe to engage in low to moderate physical exercise, (4) suitable to engage in a group intervention and (5) capable of providing written informed consent. In order to facilitate the once-off implementation of the intervention, the aim was to enrol 8–10 participants, with samples exceeding these numbers deemed clinically inappropriate.

### Intervention

The ‘Living with Multiple Myeloma Group’ consisted of six weekly group sessions, with each session being made up of a 45-min physical exercise component, a 15-min break and a 75-min structured psychosocial and self-management component. Due to the Covid-19 pandemic, the intervention was delivered online via Microsoft Teams. During the physical exercise component, educational content regarding physical exercise was delivered and participants engaged in supervised exercise, in line with individualised exercise plans devised for them at baseline. The psychosocial and self-management component was informed by cancer survivorship literature (e.g. see core self-management skills [29]), covering topics such as adjustment to diagnosis and illness, treatment side effects, relationships and meaning making while also including skills practice (e.g. stress management), self-reflection and group and pair peer discussions.

The intervention was designed and delivered by a Senior Clinical Psychologist (2nd author) and Senior Physiotherapist (4th author), with input from a Senior Oncology Nurse, all of whom specialise in working with cancer populations. Facilitating co-creation of the intervention, participants were invited to a pre-treatment focus group on the content and structure of the intervention, which six of seven invited participants attended. See Additional file 1 Table A.1. for a more detailed description of the intervention.

### Measures and materials

#### Feasibility measures

A post-treatment focus group, with a duration of 90 min, was led by the 1st author, held online and recorded via Microsoft Teams. The interview schedule addressed the acceptability and appropriateness of the intervention, experiences related to group participation and participants’ recommendations for further intervention adaptations and refinements.

The Helpful Aspects of Therapy (HAT) form [30] was used to evaluate the acceptability and appropriateness of individual group sessions and the different components of the intervention. Administered in writing, the mixed-method, self-report measure prompted participants to reflect on and describe helpful and hindering events within each of the six weekly sessions, rating each event on a 5-point scale of how helpful or hindering it was.

#### Patient-centred effectiveness measures

##### Physical outcome measures

*The Six-Minute Walk Test* was used to assess aerobic capacity and has previously been safely used in MM patients [31]. Under supervision, participants were asked to walk between two cones placed 20 m apart, at their fastest pace and covering as much distance as possible, for 6 min, with the distance covered recorded in metres.

*The Sit to Stand Test* was used to assess the functionality of the lower body and has been demonstrated to be safe in similar populations [32]. Participants sat on a bench (height 47 cm) with arms across their chest, feet flat and parallel and shoulder-width apart on the floor, and were asked to stand up and sit down 10 times as quickly as possible, fully extending the legs on each stand. The time taken to perform 10 repetitions was recorded. Participants performed three trials, with the best trial taken for analysis.

*The Grip Strength Test* was used to measure hand grip strength and has been safely administered to those with MM previously [12]. Using an electronic dynamometer, participants stood with their arm straight by their side and were asked to flex the elbow to 90° and perform 3 consecutive contractions, 30 seconds apart. The mean value was calculated.

##### Self-reported outcome measures

*The European Organisation for Research and Treatment of Cancer Core Quality of Life Questionnaire*** (**QLQ-C30) [33] was used to assess health-related QoL. The QLQ-30 is a 30-item self-report scale, comprised of multi-item and single-item subscales: the Global Health Status Scale, which provides an overall score for perceived QoL, and five functional and three symptom subscales. Each subscale is scored from 0 to 100, with a high score on the Global Health Status Scale indicating high QoL, on the functional scale indicating a healthy level of functioning, and on the symptom scale indicating high symptomology.

*The European Organisation for Research and Treatment of Cancer Multiple Myeloma module* (MY20) [34] is a supplementary module to the QLQ-C30. The MY20 is a 20-item self-report scale, incorporating three subscales (disease symptoms, side effects of treatment, future perspective and body image). It is designed to further elaborate on overall QoL assessments provided by the QLQ-30 in terms of MM-specific QoL issues. The scoring approach to the QLQ-MY20 is identical to the QLQ-C30.

*The Multidimensional Fatigue Inventory* (MFI) [35] was used to assess participants’ recent experience of fatigue. The MFI is a 20-item self-report scale, comprised of the General Fatigue Scale, which provides an overall score for perceived fatigue, and four subscales (physical fatigue, mental fatigue, reduced motivation and reduced activity), each containing 4 items. Scores for each scale are calculated by summating, ranging from 4 to 20, and higher scores indicate greater fatigue.

*The Depression Anxiety and Stress Scale* (DASS-21) [36] was used to assess symptoms of depression, anxiety and stress experienced within the previous week. The DASS-21 is a 21-item self-report scale, consisting of three subscales: depression, anxiety and stress, containing seven items each. Scores for each subscale are calculated by summation, with higher scores indicating greater levels of depression, anxiety or stress.

*A Goal Setting and Rating Scale* (GSRS) was used to evaluate goals-based outcomes (adapted with permission using the Parents Plus Client Goals Scale as a template [37]). The form encouraged participants to set an exercise, nutrition and well-being goal, with the opportunity to set two ‘other’ goals, related to what they hoped to achieve as a result of engaging in the group. Proximity to achieving each goal was rated by each participant on a scale of 0 (very far away from reaching the goal) to 10 (have reached the goal).

### Procedure

Following screening, consenting participants were referred to and contacted by the clinicians delivering the intervention, who shared further intervention details (e.g. timing, content of the intervention) over the phone and booked those able and willing to participate for individual in-person baseline assessments in the community cancer support service. After written informed consent was obtained, demographics, physical and patient-reported outcome measures were collected, the individualised exercise plans were devised by the physiotherapist and the participants received six hard copies of the HAT, which they were asked to complete at the end of each of the six online group sessions at home. One week post-intervention, participants completed physical and patient-reported outcome measures in person in the cancer support service and attended the post-intervention focus group, online. Three months after the intervention had ended, participants attended the cancer support service again, where they completed in-person follow-up physical assessments and patient-reported outcome measures and were invited to review and comment on findings from the focus group analysis.

### Data analysis

Qualitative data obtained via the focus group, the HAT and GSRS forms were analysed separately using the descriptive and interpretative approach, which has previously been used in oncology populations and lends itself to the analysis of data from various sources [38, 39]. Data was analysed jointly by the 1st author (a counselling psychologist working in psycho-oncology with experience in conducting and publishing qualitative research) and 3rd author (a masters level health psychology graduate) via the following steps: Following data preparation (verbatim transcription of the focus group recording, digitalisation of HAT and GSRS), data was cleaned and checked (e.g. entirely off topic segments from the focus group transcripts were deleted; non-specific goals on the GSRS were omitted). Domains of investigation were determined in line with the focus group interview schedule and the HAT and GSRS open-ended questions, respectively, to provide an organising structure for the data. The focus group domains were as follows: (1) perceptions and experiences related to the acceptability and appropriateness of the group; (2) helpful experiences related to group participation; (3) hindering experiences related to group participation; (4) recommendations for further developments and adaptations of the group. HAT domains were as follows: (1) helpful and (2) hindering events and (3) helpful and (4) hindering impacts that occurred during group sessions. GSRS domains were as follows: (1) exercise, (2) nutritional and (3) well-being goals. Next, all data was broken down into meaning units (i.e. individual segments of data containing a particular meaning), and within their respective domains, meaning units were then clustered into categories and subcategories according to similarities. Subsequently, findings were abstracted and underwent credibility and validity checks, which involved reflexive and iterative discussions about alternative categorisation and abstractions between the 1st and the 3rd author until a consensus was reached. Furthermore, to address potential shortfalls of the focus group (e.g. underrepresentation of non-normative perspectives) and to enhance credibility [40, 41], member checks were conducted regarding the focus group analysis at the 3-month follow-up time point, with participants being presented with a draft of the analysis and given the opportunity to comment on and rate their level of agreement with each finding. Agreement with findings was high overall (see Additional file 2, Table B.1), supportive of credible analysis. Additional data collected during member checking was integrated into the analysis during the write-up of findings.

To quantify change over time in terms of the physical measures, effect sizes were calculated from baseline to post-treatment and post-treatment to follow-up, respectively. Paired samples Cohen’s d and its 95% confidence interval (CI), utilising the non-centrality parameter method, were calculated in R using the ‘effect size’ package. For clarity purposes, data was coded so that positive effects sizes represented improvements across outcomes, and results are presented visually in a forest plot. Cohen’s d interpretation was as follows: 0.2–0.5 = small; 0.5–0.8 medium; > 0.8 = large effect [42].

Given the non-normal distribution of all patient-reported outcome measures and the small sample size, the use of already in the literature established meaningful change indices was deemed the most appropriate analytic approach for patient-reported outcomes. The respective cut-offs to determine the presence of clinically meaningful change were as follows across the measures: QLQ-C30, 5-point difference [43]; QLQ-MY20, 10-point difference for disease symptoms scale and side effects of treatment scales, 13-point difference for body image scale and 9-point difference for future perspective scale [44]; MFI: 2-point difference [45]; GSRS: 2.45-point difference (as appropriate for similar goals-based outcome tools [46]); DASS-21: cut-off scores for normal, mild, moderate, severe and extremely severe symptom levels are provided in the manual [36], with reliable change indicated by a move from one label to another. During data screening, one participant was excluded from the DASS-21 analysis, due to exhibiting clear signs of systematic and biased responding at baseline (half of the DASS-21 was answered with ‘0’, half with ‘3’, with responses directly contradicting each other). No such issues were observed regarding the remaining data.

## Results

**Fig. 1:**
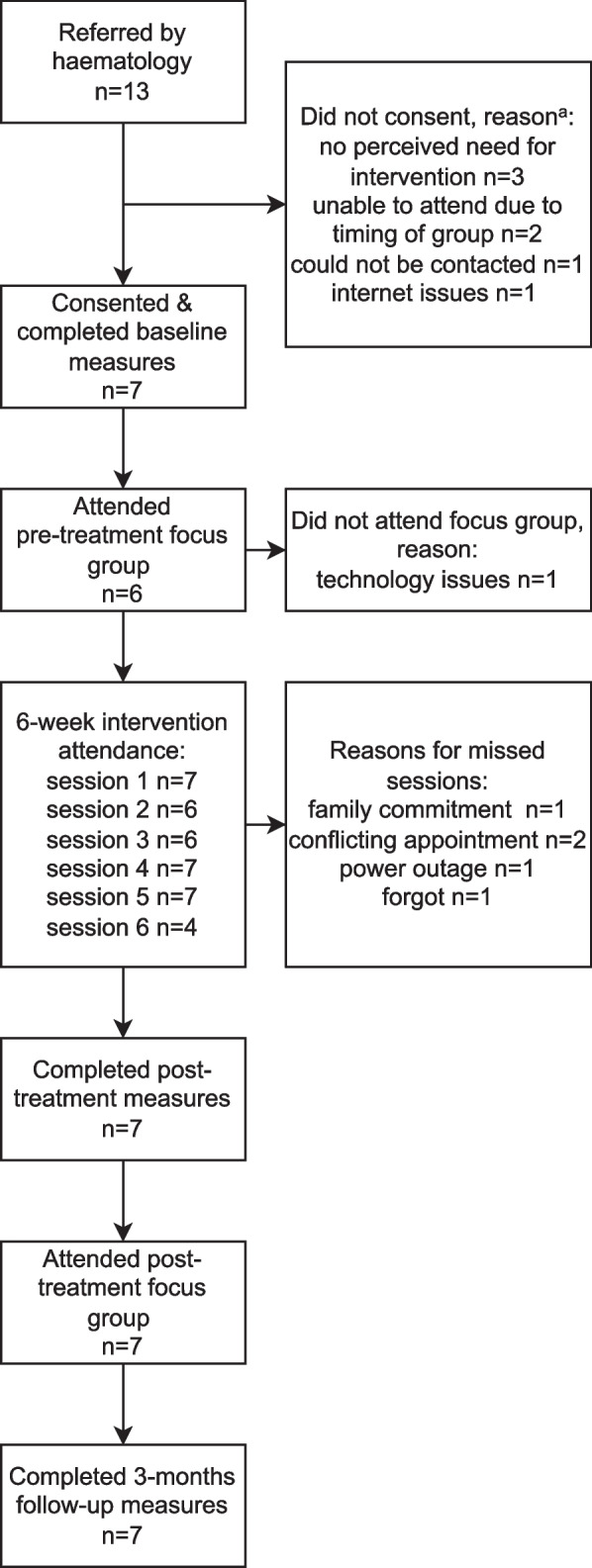
Flow chart of participation and individual session attendance rates. Note: ^a^One person indicated two reasons for not participating in the study

Thirteen participants were invited to the study by their consultant haematologist between September and October 2021, of which seven consented and commenced participation. Among these seven, five were males, and the average age was 62.86 years (*SD* = 10.07). All seven participants were married. Four were retired, two were working part-time and one was on sick leave. Four were receiving active treatment at the time of the study, and the average time since diagnosis was 44.14 months (*SD* = 24.25). All participants scored above the norm for MM patients on the QLQ-C30 Global Health Status Scale at baseline (*M* = 79.76, *SD* = 11.64; MM patient norm *M* = 55.7, *SD* = 22.8 [47]), suggesting above average health-related QoL among participants. There were no dropouts from the intervention or research and no adverse/harmful effects reported. Across all participants and scheduled sessions, 37 out of 42 were attended (see Fig. 1).

### Primary feasibility findings

#### Post-treatment focus group findings

##### Domain 1: Perceptions and experiences relating to the acceptability and appropriateness of the group

All participants (7/7) found the ‘exercise component important and satisfying’, the ‘psychosocial component engaging’, the ‘unsupervised break essential in forming peer relationships’, the ‘facilitation highly satisfying’ and the ‘involvement of their haematologist important’. While all participants (7/7) agreed that ‘online delivery of the intervention increased accessibility’, some participants (3/7) also perceived ‘online delivery as difficult at times’. Analyses suggested that the ‘group session length reduced accessibility’ and ‘individual and illness-related factors impacted group relevance’; however, participants’ views varied in this respect (regarding session length: 2/7 neither agreed nor disagreed, 5/7 agreed; regarding group relevance: 3/7 disagreed, 4/7 agreed). See Additional file 2, Table B.1., categories 1.1–1.9 for details and quotes.

##### Domain 2: Helpful experiences related to group participation

All participants (7/7) agreed that helpful experiences included the ‘invaluable companionship and peer support’ received, perceiving the group as an ‘open, safe and confidential space’, with ‘intentions to maintain continued peer support’. Most (6/7) believed their ‘physical abilities improved’, with all (7/7) wishing to ‘maintain the physical activity’ level they had achieved in the group. All (7/7) agreed that ‘revisiting parked feelings’ was a helpful experience, with most (6/7) perceiving an ‘improvement in well-being’. See Additional file 2, Table B.1., categories 2.1–2.7 for details and quotes.

##### Domains 3: Hindering experiences related to group participation

All participants (7/7) agreed that they held an ‘uncertainty in what to expect from the group’ prior to participation and that this meant ‘daunting participation’, feelings that seemed to subside as the group progressed. See Additional file 2, Table B.1., categories 3.1–3.2 for details and quotes.

##### Domain 4: Recommendation for further developments and adaptations

All participants (7/7) recommended the ‘establishment of a peer support group’, available at each milestone of the MM journey, with some (5/7) desiring ‘support for their family members’. Most participants (6/7) agreed that the ‘exercise component of the group should be further individualised and tailored’, and that ‘on-demand, recorded exercises’ would supplement this. All (7/7) recommended ‘a blend of online and in-person group delivery’ for future rollouts of the group, with the inclusion of ‘presentations from medical and well-being specialists’. Some participants (4/7) felt that the ‘group session length and timing should be adjusted’ in future rollouts. Most (6/7) recommended a ‘more detailed agenda before group sessions’, to combat uncertainty around what to expect from the group. See Additional file 2, Table B.2. for details and quotes.

#### Helpful aspects of Therapy (HAT) form findings

Across six sessions and seven participants, 37 HAT forms were completed. Helpful and hindering events and impacts associated with group participation are summarised in Table 1, with further descriptions and quotes provided in Additional file 2, Table B.2. Sixty-five helpful and three hindering events occurred during the psychosocial component and 19 helpful and three hindering events during the physical exercise component.

**Table 1:** Helpful and hindering events and impacts during group se﻿ssions participation

**Domain 1: Categories of helpful events**	**N**_**o**_	**Domain 3: Categories of helpful impacts**	**N**_**o**_
1.1. Peer discussion	7/7	2.1. Feeling connected and less alone	6/7
1.1.1. Sharing of experiences and information	7/7	2.2. Awareness and reflection	6/7
1.1.2. Sharing of emotions	5/7	2.3. Looking forward with new perspective	4/7
1.2. Physical exercise	7/7	2.4. Feeling soothed and relaxed	3/7
1.2.1. In-session physical exercise	7/7	2.5. Learning new information and skills	7/7
1.2.2. Education on physical exercise	4/7		
1.3. Psychosocial and self-management content	7/7		
1.4. Provision of nursing information and advice	5/7		
**Domain 2: Categories of hindering events**	**N**_**o**_	**Domain 4: Categories of hindering impacts**	**N**_**o**_
3.1. Peer discussion content	1/7	4.1. Experiencing unpleasant emotions	2/7
3.2. Physical exercise intensity	2/7		
3.3. Technical difficulties	1/7		

*N*_*o*_ refers to how many participants out of the total sample (*N* = 7) provided a meaning unit relevant to each category across the six group sessions

### Secondary patient-centred preliminary effectiveness findings

#### Physical outcomes

**Fig. 2:**
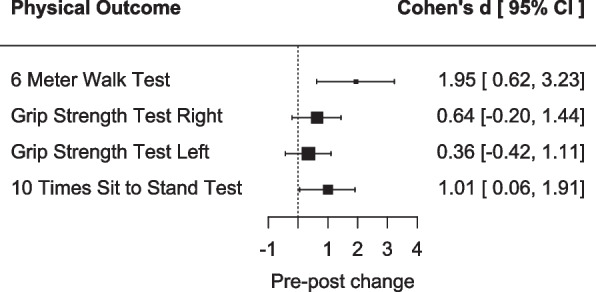
Forest plot depicting effect size of baseline to post-treatment change in physical outcomes

Standardised mean differences (i.e. Cohen’s d) suggested overall improvements across all physical outcomes at post-treatment (see Fig. 2). Largest improvements were seen in the Six-Meter Walk Test (*d* = 1.95; 95% CI 0.62, 3.23), followed by the Sit to Stand Test (*d* = 1.01; 95% CI 0.06, 1.91). However, CIs were very wide for all outcomes, limiting the reliability of all effect sizes, and crossed the zero line of no effect for both Grip Strength tests (Right: *d* = 0.64; 95% CI −0.20, 1.44; Left: *d* = 0.36; 95% CI −0.42, 1.11).

Standardised mean differences between post-treatment and follow-up measurements were indicative of a maintenance of effects at follow-up for all outcomes (6MWT: *d* = 0.16; 95%, CI −0.59, 0.90; Grip Strength Test Right: *d* = 0.17; 95%, CI −0.59, 0.91; Grip Strength Test Left: *d* = 0.60; 95%, CI −0.23, 1.39), bar the Sit to Stand Test, for which further improvement was observed (*d* = 1.40; 95%, CI 0.30, 2.45; see Additional file 2, Table B.1.)

### Self-reported outcomes

In terms of QoL, the QLQ-30 Global Health Status Scale suggested improvement for 1/7, deterioration for 2/7 and no change for 4/7 participants at post-treatment, with 2/7 showing an improvement, 2/7 a deterioration and 3/7 no change from post-treatment to follow-up. According to the MFI, general fatigue had decreased for 1/7, increased for 2/7 and remained unchanged for 4/7 participants at post-treatment, with 3/7 showing a decrease, 2/7 an increase and 2/7 no change from post-treatment to follow-up. Results for all subscales can be found in Additional file 2, Tables B.5–7. According to the DASS-21, most participants exhibited normal levels of depression (5/6), anxiety (4/6) and stress (4/6) across all time points. Regarding depression, 1/6 experienced an increase at post-treatment, which resolved to normal levels at follow-up. Regarding anxiety, 2/6 exhibited increased levels at post-treatment, with 1/6 maintaining this increase and 1/6 returning to normal levels at follow-up. Regarding stress, 1/6 experienced a decrease and 1/6 an increase in stress at post-treatment; at follow-up, one participant saw an increase in stress once again and the other maintained their post-treatment score.

Goals-based outcomes as measured by the GSRS were assessed among 20 goals named by participants and rated at each time point. Qualitative analyses of individual goals suggested 11 separate goal categories across three goal domains. Exercise domain goals related to cardiovascular fitness, strength and stretching, nutrition domain goals related to dietary changes and meal scheduling, and well-being domain goals covered a wide array of goals, from weight and functional goals to adjustment to illness, lifestyle and communication goals (see details in Additional file 2, Table.B.8). Across goal domains, 2/7 exhibited reliable improvement in their progress towards achieving at least one goal at post-intervention. Improvements observed related to exercise goals for 1/7, nutritional goals for 1/7 and well-being goals for 2/7. At follow-up, 1/7 experienced an improvement in an exercise goal, while 3/7 reported a reliable deterioration in their progress towards their goal; however, for 2/7, this deterioration still represented a reliable improvement over baseline scores. Where barriers to attaining goals were reported, these related to current physical health, e.g. undergoing surgery, and cancer treatments.

## Discussion

The primary objective of the current study was to evaluate the feasibility of a newly developed survivorship intervention for those living with MM, which was deemed good overall, while also highlighting a number of areas for development. Adoption of the intervention was promising at 54% (7/13) uptake among participants referred by their haematologist, especially in the context of previously reported poor uptake of cancer survivorship programmes [48, 49]. Regarding the intervention itself, adherence was good at 88% (37/42 of assigned sessions were attended), and participants described overall satisfaction with the content, structure and delivery of the intervention. Helpful events and impacts occurred for participants during all components of the intervention and far outweighed hindering events and impacts—speaking to the acceptability and appropriateness of the intervention. Concurrently, opportunities to further enhance acceptability (e.g. by providing an agenda to address hindering experiences in first joining the group) and appropriateness (e.g. by further individualising exercise) were reported.

The secondary objective of the study was to evaluate the preliminary effectiveness of the intervention. Here, findings were mixed. Physical outcomes were encouraging, with large effect sizes found for aerobic capacity and lower body functionality improvements (*d* = 1.01–1.95) but smaller, less reliable effect sizes for grip strength (i.e. CIs crossed into negative values, suggested possibility of null effect). Importantly though, physical gains made were maintained or improved upon at follow-up, a crucial marker for the effectiveness of exercise interventions [50]. Patient-reported outcomes were not as positive, with less than 30% of participants showing a reliable improvement on any of the measures at post-intervention (i.e. the highest reliable improvement rate was 2/7 for goals-based outcomes) and equivalent reliable deterioration rates (i.e. highest reliable deterioration rate was 2/6 for DASS-21 anxiety scale). With a similar picture emerging at follow-up, these patient-reported outcomes—providing little support for the effectiveness of the intervention—are somewhat at odds with qualitative findings, which highlighted the benefits of the intervention (e.g. invaluable peer support, a sense of connectedness, reflection, learning and improved well-being) and suggested only few and manageable hindering experiences.

Our research findings contribute to the cancer survivorship literature in several ways. Primarily designed as a multidisciplinary, self-management intervention, the most important benefits participants perceived from the intervention were related to peer support, aligning with previous research in highlighting the importance of social support in the adjustment to MM [18–20, 51]. Furthermore, as this research commenced 20 months into the Covid-19 pandemic, suggested to have exacerbated social isolation for haemato-oncology patients [52], our findings may also speak to a growing desire for peer support in the context of social distancing measures in this cohort (e.g. organic opportunities for peer support were often removed as social distancing precluded common waiting rooms).

Regarding the lack of reliable improvements in patient-reported outcomes observed in this study, a number of explanations are possible. Despite all measures being reliable, valid and previously used in similar cohorts, the timing of measurement (i.e. after physical assessments) and the substantial number of items across all measures may have impacted upon participants’ thoroughness in completing the measures and thus their reliability. Also, participants in this study did not seem to present with a particularly high symptom burden at baseline, and thus ceiling effects may have existed within individual measures (e.g. average QLQ-C30 scores were above MM patient norms [47]; majority scored in the normal range of the DASS-21 across time points). Finally, and most importantly, the intervention content and structure may have not sufficiently aligned with the patient-reported constructs measured within this study. Regarding fatigue, the timeframe of the intervention may not have been long enough, with previous research suggesting exercise interventions need to be at least 6 months long to reliably affect fatigue [12, 13]. Regarding psychosocial, self-management and peer support components of the group, constructs like adjustment, empowerment and subjective well-being (e.g. based on Foster and Fenlon’s model of recovery in cancer survivorship and social comparison theory; [53–55]) may be of greater relevance than health-related QoL, with its more medical focus on MM symptoms and treatment side effects, which naturally fluctuate as a result of frequent, intermittent and recurring MM treatments.

Still, despite the obvious need for further research to evaluate the effectiveness of the ‘Living with Multiple Myeloma Group’, participants appreciated the holistic approach taken within the intervention, linking in with prior research that has mapped out the breadth of unmet support needs within this cohort [51]. In this vein, the overall multidisciplinary content of the intervention was deemed to require relatively few adaptations. However, regarding the structure and delivery of the intervention and in line with participant recommendations, future implementations should consider (1) shortening the intervention, (2) further tailoring exercise plans by, for example, including more structured video-based home practice, (3) addressing hindering unpleasant emotions prior to and during the intervention, where possible, by providing an agenda and emphasising self-soothing, (4) blending online and face-to-face delivery of the group by including an initial face-to-face meet and greet and (5) facilitating ongoing peer support by establishing a pathway for participants to ‘graduate’ into a pure peer support group after the intervention has ended. From a service point of view [28], these suggested intervention modifications may also reduce the resource intensity of the intervention and thereby further enhance its feasibility.

With the current study establishing the overall feasibility of the intervention, future evaluations should focus on its effectiveness [26]. As such, an adequately powered, randomised controlled trial (RCT), or alternatively, a preference trial, utilising statistical measures to account for the lack of randomisation (e.g., propensity score modelling [56]), ought to be considered [26, 27]. As a first step, the execution of a pilot RCT will be advisable in informing sample-size calculations for a full power trial and addressing remaining uncertainties regarding the feasibility of the RCT in relation to methodological decisions specific to such a design (e.g. randomisation and data collection procedures). The inclusion of measurements of service user, provider and system-level implementation outcomes in this pilot RCT will be necessary to continue to ensure the suitability of the intervention for routine use [26, 28]. Finally, as discussed above, outcome measures employed in the current study may have not captured intervention effects sufficiently; hence, in future studies, delineation of primary and secondary outcomes, based on relevant theories around adjustment to cancer, self-management and peer support will be crucial.

The study has several limitations. The reliability of findings is limited by the small sample size. Regarding the quantitative findings, this meant, that due to insufficient power, we were unable to implement inferential statistics. Regarding qualitative findings, the study’s sample size limits the generalisability of findings, as saturation may not have been reached across qualitative analyses. In addition, the convenience sampling approach employed in this study may have inadvertently resulted in the selection of participants with unique characteristics and not a sample representative of the wider MM population. Also, given convenience sampling, the observed intervention uptake may not be an entirely accurate predictor of adoption in future implementations of the intervention. Finally, the 4th author was unavailable to supervise follow-up physical assessments, which were thus executed by an exercise physiologist, employed by the cancer support service, based on the 4th author’s instructions. This change in personnel may have impacted upon physical outcomes at follow-up.

In conclusion, the current study represents the first phase in the development and establishment of a survivorship intervention for those living with MM. The strengths of the study lie in its mixed methods design and multifaceted effort to elicit MM survivors’ experiences and perceptions of the intervention; ensuring their voice will be heard in informing further intervention development and research. The next phase of development will require a focus on the efficacy and effectiveness of the intervention, as without clear evidence for the effectiveness the relevance of the intervention in routine care remains unclear.

### Supplementary Information

Additional file 1. Additional Intervention Details: Intervention Group Session Structure and Content. 

Additional file 2. Additional Results: Tables and Figures. 

## Data Availability

Data that can be fully anonymised is available upon request from the first author.
