# Supplementary material for: A multidisciplinary group-based survivorship intervention for those living with multiple myeloma: a feasibility study
Source: Pilot Feasibility Stud. 2024 Jul 15;10:100. doi: 10.1186/s40814-024-01524-1 (PMC11247835; doi:10.1186/s40814-024-01524-1)
Supplement: Supplementary file 1 — Additional file 1. Additional Intervention Details: Intervention Group Session Structure and Content. [file 40814_2024_1524_MOESM1_ESM.pdf]

Additional File 1

Additional Intervention Details

Table A.1. Intervention Group Session Structure and Content

**Session No.**	**10:00am – 10:45am**<br><br>(*Led by Senior Physiotherapist*)	**10:45am – 11am**	**11am – 12:00/12:15pm**<br><br>(*Led by Senior Psychologist*)
Session 1	Introductions<br>Physical exercise & Tai Chi	Break	Psychological responses and coping with a Multiple Myeloma diagnosis.
Session 2	Education around fatigue and MM.<br>Physical exercise & Tai Chi	Break	Coping and adjustment: Stress management
Session 3	Physical exercise focused on core muscles, correct technique, and stretching.<br>Education around posture and back health	Break	Living with a chronic illness
Session 4	Physical exercise focused on core muscles, cardio and strengthening, correct technique and stretching.<br>Education around the nervous system	Break	Common treatments for Multiple Myeloma, coping with side effects and lifestyle considerations<br>(*Led by Senior Oncology Nurse*)
Session 5	Physical exercise focused on core muscles, cardio and strengthening, correct technique and stretching.<br>Education around goal setting.	Break	Body image, sexuality, relationships and communication
Session 6	Physical exercise focused on core muscles, cardio and strengthening, correct technique and stretching.<br>Education around how to continue the physical exercise journey post-intervention.	Break	Meaning making, values and participation in activities of interest
