# Supplementary material for: A multidisciplinary group-based survivorship intervention for those living with multiple myeloma: a feasibility study
Source: Pilot Feasibility Stud. 2024 Jul 15;10:100. doi: 10.1186/s40814-024-01524-1 (PMC11247835; doi:10.1186/s40814-024-01524-1)
Supplement: Supplementary file 2 — Additional file 2. Additional Results: Tables and Figures. [file 40814_2024_1524_MOESM2_ESM.pdf]

Additional File 2

Additional Results: Tables and Figures

Table B.1. Focus Group: perceptions and experiences of the 'Living with Multiple Myeloma Group' reported by participants at post-treatment and further reflected on at follow-up (N=7)

Category	Description of category [a]	No. of participants in category [b]	Member checking level of agreement [c]	Example quotes
**Domain 1: Perceptions and experiences relating to the acceptability and appropriateness of the group content, structure, and delivery**				
1.1. The exercise component as important and overall satisfying	The exercise element of the intervention was considered acceptable by the group, with engagement considered desirable for physical and mental health reasons. While the level of physical activity was deemed appropriate for the group as a whole, participants felt it would have benefitted from further tailoring.	5/7	3/7 agreed. 4/7 strongly agreed	"I think it's important that physical activity is included as a component for both mental and physical health." " I felt able for a greater physical activity level but was happy with the level deemed appropriate for the whole group."
1.2. The psychosocial component of the group as engaging	Participants described the psychosocial and self-management content as satisfying and mostly appropriate, tapping into important topics of discussion.	2/7	3/7 agreed. 4/7 strongly agreed.	"As for the content? Every week, like there was no shortage of us talking about it and contributing." "Did it match where I'm at now? It did. Yeah, it did. Several parts of it."
1.3. The unsupervised break as essential in forming peer relationships	The 10–15-minute break between the exercise and psychosocial part of the group, during which facilitators stepped away from their computers, was described as important in allowing participants to network with each other and form bonds.	7/7	1/7 agreed. 6/7 strongly agreed.	"It gave us the opportunity of networking and it was that freedom […] that allowed us to become the family that we now are" "one of the best parts of it all"
1.4. Facilitation of the group as highly satisfying	Participants reported that facilitators were well-organised, and the atmosphere was comfortable.	3/7	7/7 strongly agreed with the statement.	"They've [*the clinicians delivering the group*] done brilliantly when you consider what they've done on Zoom."

				"I felt it was very good and I think it was… that was due to [*the clinicians delivering the group*] and all the group members that left me at ease."
1.5. Hematology consultant involvement as important	Members of the group greatly appreciated the involvement of their trusted consultant in this project, for some his involvement having encouraged them to participate in the group and research in the first place.	3/7	7/7 strongly agreed with the statement.	"The one thing that encouraged me so much was to think you know, [*the participants' consultant*] took time out and to ring in person to ask you to join the group […]" "wonderful and to hear latest news, highly appreciated"
1.6. Group session length reducing accessibility of the group	Participants described sessions as potentially too long and conflicting with other commitments depending on individual circumstances (e.g., work, medical appointments). At the same time, session length suited some participants and sessions were experiences as going by quickly.	4/7	2/7 neither agreed nor disagreed. 1/7 somewhat agreed. 1/7 agreed. 3/7 strongly agreed with the statement	"I'd agree with maybe the […] two and a quarter hour or whatever is a bit long, that it could be cut to you know, an hour and a half" "I found the time flew. It's not that aspect of it, it's just finding the time in the day more so than anything else"
1.7. Online delivery of the group as increasing accessibility	Travel time and effort being eliminated, and the group proceeding despite Covid-19 were reported as benefits of the group having been delivered online. Under different circumstances, participants would have opted for a blended delivery of the group (incl. both face-to-face & remote sessions).	5/7	3/7 agreed. 4/7 strongly agreed with the statement	"If we were traveling everyday there would be an awful lot more time involved in [*group participation*]" "I felt it was easy [*engaging in the group online*]."
1.8. Online delivery as difficult at times	Difficulties some participants experienced related to trying to watch a screen while exercising, online groups being particularly tiresome and how face-to-face contact would have aided group processes.	3/7	3/7 disagreed. 1/7 neither agreed nor disagreed. 2/7 somewhat agreed. 1/7 agreed with the statement	"Perhaps it made the exercise element a bit more difficult but not a major problem" "I think if we had an initial session, we were all able to sit down and have a cup of tea. And, you know, have a chat to each other […]

				that would kind of got over that... that hurdle”.
1.9. Individual and illness related factors impacting group relevance	Some participants described other health conditions and the point in their life and on the cancer journey (e.g. post stem cell transplant) as having influenced the relevance of the group at times.	3/7	1/7 strongly disagreed. 2/7 disagreed. 1/7 somewhat agreed. 2/7 agreed. 1/7 strongly agreed with the statement	“Did it match where I'm at now? […] Some parts of it didn't obviously because I... I feel I'm maybe at a different level than some... than others. But that's natural because of the age difference in the group.” “I felt that the exercises were too long. I felt they were too stressful. But then, of course, I discovered last Tuesday that I’d an [*another health condition*].”
**Domain 2: Helpful experiences related to group participation**				
2.1. Invaluable companionship and peer support	All participants spoke about the companionship between them having been one of the most important outcomes of the group, facilitating sharing insights, learning from each other and expressing emotions.	7/7	1/7 agreed. 6/7 strongly agreed with the statement	“Yeah, the support that I've received just from being able to speak and to hear and to help and to connect; that's the best part.” “Ehm, the companionship of the group, I think, is… is everything.”
2.2. The group as an open, safe, and confidential space	Participants described appreciating and feeling reassured by the confidentiality and openness within the group.	2/7	1/7 agreed. 6/7 strongly agreed	“And everybody was so open with each other, and you know, I know it's confidential and it is confidential with me.” “I wouldn't be a man to be talking in public like or to other people, in front of other people we’ll say, and I was a bit taken aback for the first few meetings, but I must say I got very relaxed as it went on.”

2.3.	Intentions to maintain and provide peer support	Participants expressed their availability for peer support to each other as well as for others who are only beginning their journey with Multiple Myeloma.	4/7	1/7 somewhat agreed.<br>6/7 strongly agreed with the statement	"So please God, we talk again and if anyone needs anything or wants to talk here like, they know my number, I'm available, as [Pp name] says, no problem."<br>"...Multiple myeloma Ireland have support groups in many different corners of the country, but there's none in Galway and... and I don't know what people feel... if we are that group Now. You know, maybe. We can be there to lead people gently 'cause we know what not to say."
2.4.	Improvements in physical abilities	Improvements in physical abilities were reported by some participants, whereas others did not notice much change.	2/7	1/7 neither agreed nor disagreed.<br>2/7 somewhat agreed.<br>3/7 agreed.<br>1/7 strongly agreed with the statement	"We've all improved at it [the physio exercises], I think, from hearing each other."<br>"That walking and speeding yourself, speeding up during the walk. You know, and I never thought of that before, but we do that every morning now when we go walking."
2.5.	Intentions to maintain physical exercise	Intention to continue to engage in physical activities as a result of participating in the group were expressed.	2/7	3/7 agreed<br>4/7 strongly agreed with the statement	"And it's like something we'd like to continue, and I certainly will try."<br>"Sparked awareness to continue physical activity."
2.6.	Revisiting parked feelings	Participants described revisiting issues related to living with multiple myeloma during the group. Having been unsure whether this would be useful initially, they reported the results as positive in the end.	3/7	1/7 somewhat agreed.<br>2/7 agreed.<br>4/7 strongly agreed with the statement	"From the beginning when I wondered 'do I need to be talking about this every week for six weeks and is it going to bring back everything and it's going to bring up

				everything again?' I enjoyed it very much and I had no problem with any of the topics that came up." "It's actually woke me up in terms of how... what it's all about. I... I parked a lot of it, by the way, I've parked a lot of where I am. Which is a good thing"
2.7. Experiences of improved well-being.	An overall improvement in their well-being as a result of the group was endorsed by participants.	3/7	1/7 neither agreed nor disagreed.<br>3/7 agreed.<br>3/7 strongly agreed with the statement	"And it's... my wellbeing is all the better for having been able to speak about it" "[*In response to: "has the group made a difference to your wellbeing?"*] Yeah, yeah. Absolutely yeah, yeah."
**3. Hindering experiences related to group participation**				
3.1. Uncertainty in what to expect from the group.	Participants expressed that they had been unsure of what to expect of the group initially, how it was a journey into the unknown for them. This seemed to impact upon some participants more than others.	4/7	2/7 somewhat agreed.<br>2/7 agreed.<br>3/7 strongly agreed with the statement	"The one thing about it is that all of us I think just went into the unknown. We didn't know where we were going to be. We didn't know who was going to be in it. We just started. And sort of, as they say, took it on." "Whatever about any uncertainty, it soon faded when up and running"
3.2. Daunting group participation.	Participants described having daunted participation in the group initially for what it was going to bring up for them; however, these feelings wore off as the group progressed.	4/7	1/7 somewhat agreed.<br>4/7 agreed.<br>2/7 strongly agreed with the statement	"I think at the beginning I was afraid that, ehm, being in this group and revisiting everything would mean that […] the illness defines me. And I've never had that kind of mindset and I didn't want to have it. And I don't have it. And it's the

	connection between everybody now… I was afraid that that's what would happen, but it didn't.”<br>“I think once we get stuck into it, the daunting aspect of it wore away, you know.”

[a] Descriptions of categories are based on remarks made by participants during the focus group interview (n=7) as well as written feedback provided by participants during member checking at 3-months follow-up (n=7). [b] Number of participants whose remarks during the post-treatment focus group informed a category. [c] Level of agreement with the finding reported by participants during member checking at 3-months follow-up, scored on a 7-point Likert type scale.

Table B.2. Recommendation for further developments and adaptations of the ‘Living with Multiple Myeloma Group’ reported by participants at post-treatment and further reflected on at follow-up (N=7)

Category	Description of category [a]	No. of participants in category [b]	Member checking level of agreement [c]	Example quotes
1. Establish a peer support group	The need for peer support across the Multiple Myeloma journey (e.g., from diagnosis, before & after stem cell transplant, when other support dwindle) was highlighted by participants. Participants discussed their lived experiences of Multiple Myeloma and how sharing those with peers is helpful.	7/7	1/7 agreed.<br>6/7 strongly agreed with the statement	“I started off in… in… six years ago I had met nobody, and I knew it was the unknown and I would have loved to have met people that had went that journey before me, but it didn't happen anyway.”<br>“I think it's important for people who are post-transplant, maybe sort of, six months after it or something like that, that if they met people like us, who are... who have a couple years done. You know I think it would be extremely helpful.”
2. Tailor and further individualise the exercise component of the intervention.	Participants suggested a further tailoring of the exercise element to individuals’ abilities and needs (e.g., personalised exercise plans developed at the beginning of the group). This was seen as a way of shortening the length of sessions also.	3/7	1/7 neither agreed nor disagreed.<br>2/7 agreed.<br>4/7 strongly agreed with the statement	“If you had a group once per week that we all do the same, but that there would be some tailored aspect to the program in terms of physical activity.”<br>“And perhaps at that same time [*as the initial assessment*] there's somebody from a sports science background, who could set up an actual individual schedule for each person.”
3. To include on-demand video-based tailored exercise.	It was suggested that exercise videos could be shared with participants, allowing them to access appropriate exercises for their individual needs at a time of their choosing. This was recommended to supplement rather than replace the joint exercise sessions, albeit allowing those sessions to be shorter then.	2/7	1/7 neither agreed nor disagreed.<br>2/7 somewhat agreed.<br>2/7 agreed.<br>2/7 strongly agreed with the statement.	“Having a video of exercises, I think would be a tremendous help.”<br>“[…] maybe if some of that was a recorded exercise session or whatever, that the timescale might be an hour or an hour and 20 minutes or whatever”

4. Adjust group session length and timing	Some participants felt that the length and timing of group sessions may be problematic for future cohorts, depending on individual circumstances (e.g., work, other appointments).	3/7	1/7 disagreed.<br>1/7 neither disagreed nor agreed.<br>4/7 agreed with the statement.	"I could see that people might have a problem with [*length of sessions*], but if it was to be just an hour or an hour and 20 minutes or whatever."<br>"But reducing the time span on this would be a benefit, definitely."
5. Blend online and in person delivery of the group	While online delivery was seen as facilitating accessibility of the group, it was suggested to "top and tail" the group with in-person meetings to for example support bonding.	3/7	1/7 somewhat agreed.<br>6/7 strongly agreed with the statement.	"I think if things were normal, ehm, probably a mix, maybe of the first meeting in person…"<br>"Maybe top and tail it with in-person […] and to have it online."
6. Provide more detailed agenda before group sessions	Some participants suggested that receiving more information and/or a more detailed agenda before each group session would have been helpful. However, at member checking other participants felt this would only help so much, and they did not want to have all information upfront.	2/7	1/7 neither disagreed nor agreed.<br>2/7 somewhat agreed.<br>1/7 agreed.<br>3/7 strongly agreed with the statement.	"We didn't know what we were letting ourselves in for, whereas if there was an agenda ahead of each meeting saying what's going to happen at the meeting. You know, a days' notice before hand, at least you'd know what you're letting yourself in for."<br>"Even with advance agenda I feel there would still be unknowns and therefore some apprehension"
7. Offer support to family members of those living with Multiple Myeloma	Participants described support needs in their families and loved ones, with some being in favour of a peer support group for families of individuals living with Multiple Myeloma.	5/7	2/7 neither agreed nor disagreed.<br>2/7 agreed.<br>3/7 strongly agreed with the statement	"Something that was also mentioned among ourselves […] was the point of view that there is support needed for families, not just for us"<br>"So perhaps this support group […] it could... could be utilized, as well to help in that area. Of the family I mean."
8. Include presentations by medical and wellbeing specialists.	The importance of covering physical as well as mental health aspects of living with Multiple Myeloma was described and the inclusion of specialists in the area (e.g., haematology consultant, dietician) was recommended.	2/7	1/7 somewhat agreed.<br>2/7 agreed.<br>4/7 strongly agreed with the statement	"And if you're doing another session, it should be done with [*the haematology consultant*]."<br>"More up-to-date info on the importance of food would be appreciated"

[b] Descriptions of recommendations are based on remarks made by participants during the focus group interview (n=7) as well as written feedback provided by participants during member checking at 3-months follow-up (n=7).

Table B.3. Instances of helpful and hindering events and impacts among participants who took part in the Living with Multiple Myeloma Group (n=7).

Category Name	Category Description	Examples Quotes	Enumeration Category [(a)]
**Domain 1: Helpful Events**			
1.1. Peer Discussion			
1.1.1. Sharing of experience and information	Helpful events related to participants sharing their MM experience with each other.	“Discussion among participants where valuable information was shared.”<br>“Really good to hear another's story/experience.”	General
1.1.2. Sharing of emotions	Helpful events related to participants sharing their emotions with each other.	"Open discussion on all the different range of emotions you go through."<br>“Hearing how other people's emotions are similar to mine”	Typical
1.2. Physical activity			
1.2.1. In-session physical activity and exercises	Helpful events related to performing exercises, in-session.	“I found the Tai Chi very helpful.”<br>“Stretching was very helpful also.”	General
1.2.2. Education on physical activity	Helpful events related to the information delivered by the physiotherapist regarding physical activity.	“Discussion around posture and breathing exercises”.	Typical
1.3. Psychosocial and self-management content	Helpful events related to the delivery of psychosocial and self-management content.	“Practical tips on how to manage stress and to be kind to ourselves”.<br>“[Psychologist’s name] talk in general about how cancer can affect you, family and friends.”	General
1.4. Provision of nursing information and advice	Helpful events related to nursing input around MM treatments, coping with side effects and lifestyle considerations.	“She also gave us good information about diet and exercise.”	Typical
**Domain 2: Helpful Impacts**			
2.1. Feeling connected and less alone	Helpful impacts of feeling less alone due to meeting peers, particularly where peer support was not available before.	“When I was diagnosed with myeloma, I didn’t know anybody with it. Now I can share it with 6 or more people.”<br>“I am not alone!”	General
2.2. Awareness and reflection	Helpful impacts of self-reflection and self-awareness facilitated by group participation.	“I found it [discussion around nervous system] helpful as it helped me to recognise when I am in a stressed state that it	General

		can have a negative impact on my body."<br>"My own realisation of the stories igniting anxiety and sadness in me."	
2.3. Looking forward with new perspective	Helpful impacts of viewing the future from a fresh perspective, e.g., feeling reassured, motivated, hopeful, and more confident.	"It confirmed for me that the treatment works and removed some of the doubt about the road ahead."<br>"It gave me the confidence to approach people in my life to try explain what I needed."	Typical
2.4. Feeling soothed and relaxed	Helpful impacts of feeling soothed and relaxed in the moment, during sessions.	"Had a tough week and a number of work issues were difficult, and it helped in relaxing."<br>"For some reason, it [tai-chi exercise] felt as if I was pulling positive energy back to my body and it felt soothing."	Variant
2.5. Learning new information and skills	Helpful impacts of receiving new information and learning new skills.	"Learning exercises that should be useful to help the body."<br>"I learned a lot about coping with these issues."	General
**Domain 3: Hindering events**			
3.1. Technical difficulties	Hindering events related to the group's online delivery.	"Background noise!"	Unique
3.2. Physical activity intensity	Hindering events in which exercise intensity was experienced as excessive due to individual reasons.	"As I was just out of hospital and did not exercise for two weeks, it pinched a bit."	Variant
3.3. Peer discussion content	Hindering event related to discussion around a frightening treatment-related experiences.	"[Discussing] T-cells transplant for the 2nd time."	Unique
**Domain 4: Hindering impacts**			
4.1. Experiencing unpleasant emotions	Participants described experiencing mildly unpleasant emotions, triggered by the group content.	"Just hearing some stories evokes emotions in me that weren't necessarily positive, i.e., I felt slightly more anxious, but these are far outweighed by the positive."	Variant

Note. (a) *Enumeration Category* refers to the label given to categories depending on the frequency of participants in each category across time (six sessions), i.e., general (80%), typical (50%), variant (greater than 2 participants), unique (1 participant).

Table B.4. Forest plot depicting effect sizes of post-treatment to follow-up change in physical outcomes

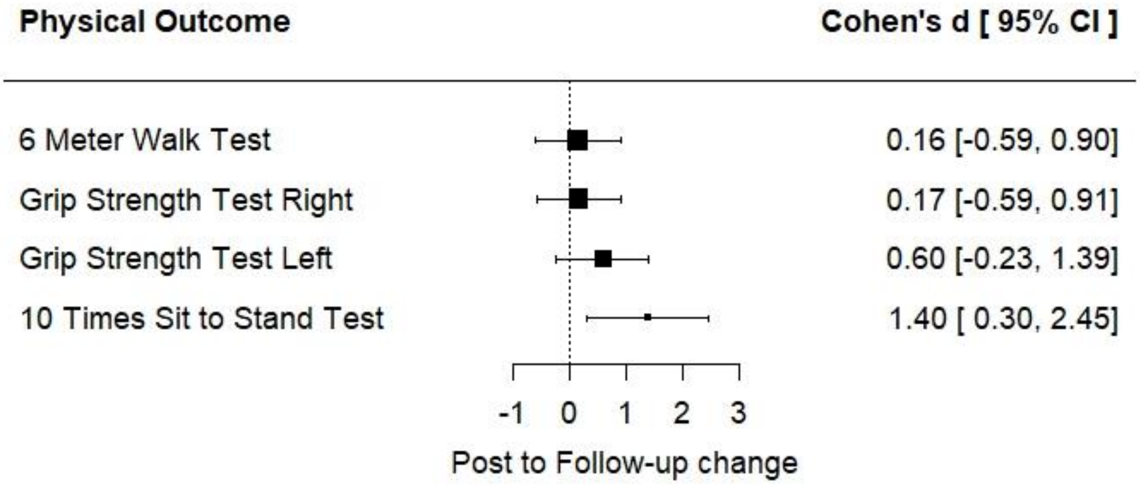

Table B.5. Reliable change on the QLQ-C30 subscales.

	Pre-Post			Post-FU		
**Function Scales[1]**	**Improvement (X/7)[3]**	**Deterioration (X/7)**	**No Change (X/7)**	**Improvement (X/7)**	**Deterioration (X/7)**	**No Change (X/7)**
Physical functioning	0/7	5/7[4]	2/7	4/7	0/7	3/7
Role functioning	1/7	2/7	4/7	2/7	1/7	4/7
Emotional functioning	3/7	1/7	3/7	3/7	2/7	2/7
Cognitive functioning	1/7	2/7	4/7	2/7	0/7	5/7
Social functioning	1/7	3/7	3/7	4/7	0/7	3/7
**Symptom Scales[2]**	**Increase (X/7)**	**Decrease (X/7)**	**No Change (X/7)**	**Increase (X/7)**	**Decrease (X/7)**	**No Change (X/7)**
Fatigue	2/7	1/7	4/7	1/7	3/7	3/7
Nausea and vomiting	0/7	0/7	7/7	0/7	0/7	7/7
Pain	3/7	0/7	4/7	1/7	3/7	3/7
Dyspnoea	1/7	0/7	6/7	0/7	1/7	6/7
Insomnia	1/7	1/7	5/7	0/7	0/7	7/7
Appetite loss	1/7	0/7	6/7	0/7	1/7	6/7
Constipation	0/7	0/7	7/7	3/7	1/7	3/7
Diarrhoea	0/7	1/7	6/7	1/7	0/7	6/7
Financial difficulties	1/7	0/7	6/7	1/7	1/7	5/7

[1]EORTC QLQ-C30 function scales: An improvement indicated better QoL, with an MCID of 5 points relevant; [2]EORTC QLQ-C30 symptom scale: An increase indicated a reduction in QoL with an MCID of 5 points relevant. [3]Results are presented in terms of how many participants out of the total sample (X/7) reported a change on each scale. [4]This unusual finding may reflect the participants' increased awareness of their physical limitations as a result of participating in the group. It is important to note that all who experienced a deterioration at post-intervention returned to baseline or improved at follow-up.

Table B.6. Reliable change on the QLQ-MY20 subscales.

	Pre to Post			Post to Follow-up		
**QLQ-MY20 Function Subscales**[1]	**Improvement (X/7)**[3]	**Deterioration (X/7)**	**No Change (X/7)**	**Improvement (X/5)**	**Deterioration (X/5)**	**No Change (X/5)**
Future Perspective	2/7	2/7	3/7	3/7	1/7	1/7
Body image	0/7	2/7	5/7	0/7	1/7	4/7
**QLQ-MY20 Symptom Scales**[2]	**Increase (X/7)**	**Decrease (X/7)**	**No Change (X/7)**	**Increase (X/7)**	**Decrease (X/7)**	**No Change (X/7)**
Disease Symptoms	1/7	1/7	5/7	1/7	1/7	5/7
Side Effects	0/7	1/7	6/7	1/7	1/7	5/7

[1]EORTC QLQ-MY20 function scales: An improvement indicated better QoL, with an MCID of 10 points relevant; [2]EORTC QLQ-MY20 symptom scale: An increase indicated a reduction in QoL with an MCID of 13 points for body image and an MCID of 9 points for future perspectives. [3]Results are presented in terms of how many participants out of the total sample (X/7) reported a change on each scale (X/7 for Function Scales Pre-Post and Symptom Scales, X/5 for function scales post-follow-up).

Table B.7. Reliable change on the MFI subscales.

	Pre-Post			Post-FU		
**MFI Subscales**[1]	**Decrease (X/7)**[2]	**Increase (X/7)**	**No Change (X/7)**	**Decrease (X/7)**	**Increase (X/7)**	**No Change (X/7)**
Physical Fatigue	4/7	0/7	3/7	2/7	1/7	4/7
Reduced Activity	2/7	2/7	3/7	2/7	2/7	3/7
Reduced Motivation	3/7	3/7	1/7	1/7	1/7	5/7
Mental Fatigue	3/7	4/7	0/7	3/7	2/7	2/7

[1]Multidimentional Fatigue Inventory subscales: An MCID of 2 points relevant; [2]Results are presented in terms of how many participants out of the total sample (X/7) experienced a change on each scale.

Table B.8. Reliable change on goal-based outcomes, measured by an adapted goal setting and rating scale.

			Pre to post				Post to Follow-up			
Goal Domain	In domain	Goal Category	In category	Improved[1]	Deteriorated[2]	No change[3]	In cateogry[4]	Improvement[5]	Deterioration[6]	No change[7]
Exercise Goals	6/7	To improve cardiovascular fitness	6/7	2/4	0/4	4/4	4/6	1/4	0/4	3/4
		To increase strength	2/7	0/2	0/2	2/2	2/2	0/2	0/2	2/2
		To improve stretching	2/7	1/2	0/2	1/2	2/2	0/2	1/2	1/2
Nutrition Goals	5/7	To reduce intake of a food group	2/7	0/2	0/2	2/2	2/2	0/2	1/2	1/2
		To increase intake of food group	1/7	0/1	0/1	1/1	1/1	0/1	0/1	1/1
		To schedule meals	2/7	1/2	0/2	1/2	2/2	0/2	1/2	1/2
Wellbeing Goals	5/7	To be present in the moment	1/7	0/1	0/1	1/1	1/1	0/1	0/1	1/1
		To maintain functionality	2/7	0/2	0/2	2/2	1/2	0/1	0/1	1/1
		To engage in leisure activities	2/7	1/2	0/2	1/2	0/2	0	0	0
		To learn to live with a chronic disease	2/7	0/2	0/2	2/2	0/2	0	0	0
		To improve communication about illness	1/7	0/1	0/1	1/1	1/1	0/1	0/1	1/1

	To maintain or lose weight	3/7	2/3	0/3	1/3	3/3	0/3	2/3	1/3

[1]Improvement at post-intervention assessment. [2]Deterioration at post-intervention assessment. [3]No change at post-intervention assessment. [4]Data provided by participants on each goal at follow-up assessment. [5]Improvement at follow-up assessment. [6]Deterioration at follow-up assessment. [7]No change at follow-up assessment.
